# Different coating application methods: Zein‐based edible coating containing *Heracleum persicum* essential oil for shelf‐life enhancement of whey‐less cheese

**DOI:** 10.1002/fsn3.4269

**Published:** 2024-06-10

**Authors:** Hadis Rajaei Lak, Behnaz Bazargani‐Gilani, Mostafa Karami

**Affiliations:** ^1^ Department of Food Hygiene and Quality Control, Faculty of Veterinary Medicine Bu‐Ali Sina University Hamedan Iran; ^2^ Department of Food Science and Technology, College of Food Industry Bu‐Ali Sina University Hamedan Iran

**Keywords:** cheese, coating methods, Golpar essential oil, Zein edible coating

## Abstract

In this research, the efficiency of brushing (Br), dipping (Di), spraying (S), and enrobing (En) methods was compared in three concentrations of 10%, 15%, and 20% of corn zein (Z) edible coating containing 0.5% of *Heracleum persicum* essential oil (HEO) in the shelf‐life improvement of whey‐less cheese during 56 days of cold storage. The results of the photography and colorimetric (L*, a*, and b* parameters) of the samples showed that the En method in 20% of Z created a uniform, brilliant, and attractive surface on the cheese pieces compared to the other groups during the storage period, and the S, Br, and Di methods were in the next categories, respectively. The findings of the texture analysis of the samples showed that all of the treatments significantly (*p* ≤ .05) preserved the hardness of the cheese samples compared to the control group, and the En method containing Z 20% and HEO was the most effective treatment in preventing the hardness loss of the samples during the 56‐day storage period. In all treatments, the growth of aerobic mesophilic bacteria, psychrotrophic bacteria, *enterobacteriaceae*, molds, and yeasts was significantly (*p* ≤ .05) reduced in comparison with the control sample, and the En method containing HEO and Z 20% was the most efficient in preventing the microbial growth. The rate of moisture loss, fat oxidation, and pH values of the studied samples significantly (*p* ≤ .05) decreased in the coated treatments containing a higher concentration of Z and HEO compared with other treatments during the storage period. According to the findings of this study, it can be concluded that the En technique containing Z20% and HEO0.5% was the most effective treatment in the shelf‐life improvement of whey‐less cheese during 56 days of the refrigerated storage period, and the S, Br, and Di methods were in the next ranks, respectively.

## Graphical abstract

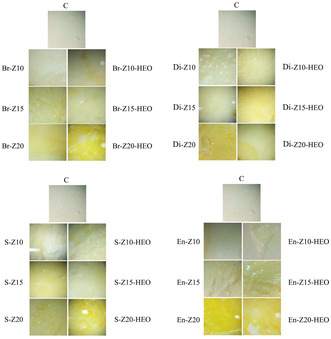

## 1 INTRODUCTION

Cheese is the most diverse category of milk products, with different shapes, textures, and flavors due to its different production and ripening methods. The special composition of cheese and the handling and storage conditions often cause the excessive growth of molds, yeast, and bacteria on its surfaces, which leads to a quality and economic loss for producers and consumers (Esparvarini et al., 2022). Whey‐less cheese is produced by adding whey protein or milk concentrate to the fresh milk, which has lower production costs compared to the other cheeses besides its high quality. Therefore, the production of this type of cheese is increasing compared to the others (Omrani Khiabanian et al., 2020). Considering the importance of the nutritional value of cheese for consumers, increasing shelf‐life and preserving the quality of this product are important issues for food producers. The use of active edible coatings for food preservation is an appropriate approach that has been recently considered in the food industry. The biopolymers are the main components of these coatings, which have been studied in various research. Zein is a biopolymer of protein origin that is produced by corn byproducts in processing plants. One of the by‐products of corn starch synthesis by corn gluten is zein protein, which is considered as a component of corn prolamin (Barkhordari & Bazargani‐Gilani, 2021; Yıldırım‐Yalçın et al., 2021). Zein coating creates an efficient barrier against various gases and therefore can delay the microbial and physicochemical changes in the coated food during the storage period. Glutamic acid, proline, leucine, and alanine are the main substituent amino acids in corn zein (Cui et al., 2020; Zhan et al., 2020). Incorporating antioxidant and antimicrobial compounds, such as herbal essential oils and extracts, in the zein matrix can impressively fortify its preservative features in the coated food. *Heracleum persicum* from the Apiaceae family is a flowering plant that is known as a popular, desirable, and common side dish, used in various foods, such as fruits, pickles, salads, and various cheeses, among people. The fruit of *Heracleum persicum* is high‐yield in essential oil production, and the produced essential oil also has strong antioxidant and antimicrobial properties (Majidi & Sadati Lamardi, 2018; Pirhayati et al., 2018). Therefore, boosting the zein edible coating with *Heracleum persicum* essential oil can provide appropriate active packaging in whey‐less cheese with pleasant sensory characteristics. The coating method can impressively affect the preservative properties of the used biopolymer in food and also the final price of the coated product. There are a few studies about the comparison of features and efficiencies of different coating methods in the shelf‐life improvement of food models. Dipping technique is the most common coating method that can be related to simplicity, ease of use, and affordability. But rapid quality loss of coating solution due to the decrease in biopolymer concentration and food residues is the main defect of this technique that can lead to the microorganisms' growth. The enrobing method is one of the coating techniques that is widely used in the chocolate industry (Zhong et al., 2014). The coating solution is poured vertically on the product surface by a nozzle with a certain diameter and distance. Spraying and brushing are other coating methods that create a uniform layer with a specified thickness on the food surfaces, with the possibility of consecutive applications without contaminating the coating solution. Choosing an appropriate coating method is not only effective in improving the preservative properties of the formed coating on food surfaces but also affects its production and operation costs (Wang et al., 2023; Zhong et al., 2014). Therefore, we intended to compare the different coating methods, such as dipping, enrobing, spraying, and brushing in different concentrations of corn zein edible coating containing HEO, in the shelf‐life enhancement of the cold‐stored whey‐less cheese in this study.

## 2 MATERIALS AND METHODS

### 2.1 Materials

Zein, methanol, ethanol, butylated hydroxytoluene (BHT), thiobarbituric acid (TBA), sodium hydroxide, glycerol, tween 80, copper sulfate, potassium sulfate, sulfuric acid, phenolphthalein, trichloroacetic acid, magnesium oxide, boric acid, violet red bile glucose agar (VRBA), plate count agar (PCA), and peptone water were purchased from Merck Company.

### 2.2 HEO extraction and analysis

*Heracleum persicum* fruits were procured from the local market in Hamedan, Iran. The fruits were ground by an industrial mill (Best, Tehran, Iran), and then the Clevenger set (Simax, Pyrexfan, Tehran, Iran) was used for hydro‐distillation for 3 h (Dini et al., 2022). After the dehydration of the obtained HEO using anhydrous sodium sulfate, the HEO components were identified by a gas chromatograph‐mass spectrometer (GC‐ MS) (Hewlett‐Packard).

### 2.3 Providing zein coating solutions

Zein solutions were prepared in three concentrations of 10%, 15%, and 20%, each containing 2% of glycerol as a plasticizer. The amount of 0.5% of HEO was added to the related solutions (Barkhordari & Bazargani‐Gilani, 2021).

### 2.4 Designing the treatments

The fresh whey‐less cheese was provided from a dairy plant in Hamedan, Iran. After transporting the samples to the laboratory under cold and sterile conditions, they were cut into 25‐g pieces and coated in the prepared solutions by four techniques of brushing, dipping, spraying, and enrobing. The designed treatments are represented in Table 1.

**TABLE 1:** Designed treatments.

Treatments				
C (control)	Brushing	Dipping	Spraying	Enrobing
Uncoated	Br‐Z10	Di‐Z10	S‐Z10	En‐Z10
	Br‐Z10‐HEO	Di‐Z10‐HEO	S‐Z10‐HEO	En‐Z10‐HEO
	Br‐Z15	Di‐Z15	S‐Z15	En‐Z15
	Br‐Z15‐HEO	Di‐Z15‐HEO	S‐Z15‐HEO	En‐Z15‐HEO
	Br‐Z20	Di‐Z20	S‐Z20	En‐Z20
	Br‐Z20‐HEO	Di‐Z20‐HEO	S‐Z20‐HEO	En‐Z20‐HEO

Abbreviations: Br, Brushing method; C, control; Di, Dipping method; En, Enrobing method; HEO, *Heracleum persicum* essential oil; S, Spraying method; Z, Zein.

### 2.5 Dipping method

In the dipping method, each cheese piece was immersed in the coating solutions for 20 s and dried on the sterile net (Wang et al., 2023).

### 2.6 Brushing method

The brushing method was performed with a sterile brush so that a layer was drawn on all the surfaces of the cheese pieces. After drying the first layer, the second and third layers were also created on the samples according to the mentioned instructions (Wang et al., 2023).

### 2.7 Enrobing method

For the enrobing method, the related coating solution was pumped up at a speed of 50 mL/min and vertically poured on the samples by a nozzle with a diameter of 6 mm and a distance of 2 cm above the samples. After the complete covering of the cheese pieces, they were dried and packaged (Zhong et al., 2014).

### 2.8 Spraying method

The spraying method was performed by a sprayer nozzle so that the coating solutions were sprayed on all sample surfaces. Feeding pressure and spray rate were considered at 1.8 kg/cm^2^ and 3.8 L/h, respectively (Wang et al., 2023).

Then, all dried‐coated samples were packaged in zip packs and stored in refrigerator conditions, and their microbial, chemical, and physical features were analyzed at 14‐day intervals during the 56‐day storage period.

### 2.9 Microbial analysis

Total viable count and psychrotrophic bacteria were enumerated on the Plate Count Agar by surface spreading culture at 37°C and 7°C for 24 h and 10 days, respectively. *Enterobacteriaceae* were enumerated on the VRBA by pour‐overlay culture at 37°C for 24 h. The population of yeast molds was determined on the Rose Bengal Chloramphenicol (RBC) Selective Agar by surface spreading culture at 25°C for 3–5 days (Yousef et al., 2022).

### 2.10 Chemical analysis

#### 2.10.1 pH

10 g of the cheese pieces was mixed with 50 mL of distilled water. After homogenizing the obtained mixture for 5 min, the solution was adjusted to 150 mL with distilled water under heating (40°C) and stirring. Then, the obtained solution was centrifuged (3000 *g* for 10 min), and next, paper filters were used for filtering. Finally, pH values of the filtrates were recorded by a pH meter (AOAC, 2012).

#### 2.10.2 Titratable acidity

10 g of the cheese pieces was mixed with 50 mL of distilled water. After homogenizing the obtained mixture for 5 min, the solution was adjusted to 150 mL with distilled water under heating (40 C) and stirring. Then, the obtained solution was centrifuged (3000 *g* for 10 min), and next, paper filters were used for filtering. Then, 25 mL of the filtrate containing 4–5 drops of phenolphthalein (1%) was titrated with sodium hydroxide (0.1 N) until the appearance of the stable pink color. TA was determined by the following formula:

$$\mathrm{TA}=\frac{a\times b}{c}\times 100$$

where *a* is the concentration of sodium hydroxide, *b* is the consumed volume of sodium hydroxide, and *c* is the weight of the studied cheese piece (AOAC, 2012).

#### 2.10.3 Lipid

The Gerber method was used for measuring the fat content of the studied samples (AOAC, 2012).

#### 2.10.4 Lipid oxidation

The thiobarbituric acid reactive substances (TBARS) technique was used for determining the lipid oxidation of the studied cheese pieces (Ünalan et al., 2013). TBARS values were reported as mg of malondialdehyde (MDA) per kg of cheese. A calibration curve was provided using 1,1,3,3‐tetraethoxypropane (TEP).

#### 2.10.5 Protein

The protein content of the cheese pieces was determined by the Kjeldahl method (AOAC, 2012).

#### 2.10.6 Moisture

The drying method by vacuum oven (Fan Azma Gostar, Tehran, Iran) was used for moisture measurement. The weight difference between the wet and dried samples at 70°C was considered to calculate their moisture content (AOAC, 2012).

#### 2.10.7 Ash

The samples were incinerated in an electric furnace (Fan Azma Gostar, Tehran, Iran) at 550°C, and their ash content was calculated by the following equation (AOAC, 2012):

$$\text{Total}\;\mathrm{ash}\;\left(\%\right)=\frac{\text{Weight of the obtained}\;\mathrm{ash}}{\text{Weight of the sample}}\times 100$$

### 2.11 Physical analysis

#### 2.11.1 Colorimetric analysis

A portable colorimeter (Minolta Chroma, Osaka, Japan) was used for the determination of the color factors of the treated samples, including a* (red/green), b* (yellow/blue), and L* (brightness/darkness) in the studied samples (Zolfaghari et al., 2023).

#### 2.11.2 Photography of the samples

Photography of the sample surfaces was performed by an optical stereo microscope (SZ61 Olympus, Tokyo, Japan) at a magnification of 13×.

#### 2.11.3 Texture evaluation

The cheese hardness was measured by a texture analyzer (STM‐Santam, UK) and a penetration test with a depth of 3 mm (Esparvarini et al., 2022).

### 2.12 Sensory analysis

A total of 20 students (10 females and 10 males, 20–30 years old) from the Food Hygiene and Quality Control Department of BU‐Ali Sina University were chosen as panelists for sensory evaluation of the treatments. After separating the used coating from the samples, a 10‐g piece of each cheese sample was tested by the panelists. A 5‐point hedonic scale was used to evaluate the taste (1: Extremely undesirable, 5: Extremely great), odor (1: Extremely unacceptable/off‐odor, 5: Extremely pleasant), and overall acceptability (1: Extremely unacceptable, 5: Extremely pleasant) (Esparvarini et al., 2022).

### 2.13 Statistical analysis

All experiments were performed in triplicate. The obtained data were illustrated as mean values ± standard deviations (SD). Variance analysis (ANOVA) with the Tukey test was used at the significance level of *p* ≤ .05 to compare differences among the studied groups using SPSS software (IBM SPSS Statistics 21).

## 3 RESULTS AND DISCUSSION

### 3.1 HEO analysis

HEO ingredients are presented in Table 2. According to the obtained results, 13 compounds in 99.01% of total HEO were identified. The main ingredients of HEO were hexyl ester butanoic acid (27.234%), 3,7‐dimethyl‐1,6‐octadien‐3‐ol (17.697%), and octyl ester acetic acid (17.320%), respectively. Past research reported octyl acetate, hexyl 3‐methylbutanoate, hexyl 2‐methylbutanoate, and pentyl‐cyclopropane as major substances of HEO with different values. These inconsistencies can be linked to the harvesting season, herb age, soil, climate, geographical position, plant drying process, and extraction procedure (Abdollahi et al., 2022; Majidi & Sadati Lamardi, 2018).

**TABLE 2:** Biochemical constituents of HEO.

Rank	Compound name	Area (%)	RT^a^
1	Dimethyl‐silanediol	2.878	4.943
2	1‐Hexanol	5.096	6.379
3	3‐Methyl‐, 1‐methylethyl ester butanoic acid	4.194	7.217
4	Butyl ester Butanoic acid	4.554	10.726
5	Hexyl ester Acetic acid	2.606	11.317
6	1‐Octanol	5.457	13.376
7	3,7‐Dimethyl‐1,6‐Octadien‐3‐ol	17.697	14.169
8	Hexyl ester Butanoic acid	27.234	16.964
9	3‐Octen‐2‐ol	5.363	17.099
10	Octyl ester Acetic acid	17.320	17.537
11	2‐Methyl‐, octyl ester Propanoic acid	2.130	21.270
12	Octyl ester Butanoic acid	2.359	22.383
13	2‐Methyl‐, octyl ester Butanoic acid	3.123	23.550
Total identified (%)		99.01	

^a^ Retention time.

### 3.2 Microbial analysis

#### 3.2.1 Total viable count

The changes in the total viable count of the studied cheese pieces are presented in Table 3. There were no significant differences (*p* ≥ .05) in the initial numeration of TVC among the studied treatments. Generally, by increasing the storage period, TVC showed an ascending trend in all treatments. The control group (uncoated cheese pieces) significantly (*p* ≤ .05) showed higher TVC than the others during storage time. The lowest TVC was related to the enrobing method, and spraying, brushing, and coating techniques were in the next ranks, respectively. Also, the highest concentration of zein (20%) containing HEO (Z20‐HEO treatment) showed the lowest TVC in each method. In other words, the effect of zein edible coating on lowering TVC was dose‐dependent. Pena‐Serna et al. (2016) used the brushing method for the zein coating of the cheese samples during the 56‐day storage period. They concluded that the cheese slices with zein coatings showed no microbiological contamination after 50 days, but the unpackaged slices were contaminated after 21 days.

**TABLE 3:** Total viable count (A), psychrotrophic bacteria (B), *enterobacteriacea* (C), and yeast‐molds (D) population of the cold stored cheese samples.

(A)						
Total viable count (log CFU/g)						
Methods	Treatments	0	14	28	42	56
C	C	0.00 ± 0.00^A^	4.27 ± 0.00^A^	4.45 ± 0.00^A^	5.59 ± 0.03^A^	5.93 ± 0.01^A^
Brushing	Z10	0.00 ± 0.00^A^	4.16 ± 0.00^B^	4.35 ± 0.01^B^	5.52 ± 0.01^AB^	5.70 ± 0.06^B^
	Z10‐HEO	0.00 ± 0.00^A^	4.07 ± 0.00^C^	4.37 ± 0.01^B^	5.45 ± 0.09^B^	5.65 ± 0.08^C^
	Z15	0.00 ± 0.00^A^	4.11 ± 0.01^B^	4.36 ± 0.06^B^	5.44 ± 0.02^B^	5.62 ± 0.04^C^
	Z15‐HEO	0.00 ± 0.00^A^	4.06 ± 0.02^C^	4.33 ± 0.01^B^	5.42 ± 0.09^B^	5.55 ± 0.02^D^
	Z20	0.00 ± 0.00^A^	4.01 ± 0.01^C^	4.24 ± 0.013^C^	5.32 ± 0.08^C^	5.52 ± 0.08^D^
	Z20‐HEO	0.00 ± 0.00^A^	3.96 ± 0.01^CD^	4.19 ± 0.00^CD^	5.26 ± 0.07^D^	5.48 ± 0.04^DE^
Dipping	Z10	0.00 ± 0.00^A^	4.27 ± 0.02^A^	4.45 ± 0.01^A^	5.59 ± 0.09^A^	5.74 ± 0.05^B^
	Z10‐HEO	0.00 ± 0.00^A^	4.27 ± 0.00^A^	4.41 ± 0.12^A^	5.49 ± 0.04^B^	5.69 ± 0.02^C^
	Z15	0.00 ± 0.00^A^	4.27 ± 0.00^A^	4.43 ± 0.00^A^	5.44 ± 0.09^B^	5.65 ± 0.07^C^
	Z15‐HEO	0.00 ± 0.00^A^	4.19 ± 0.00^B^	4.39 ± 0.01^AB^	5.42 ± 0.03^B^	5.61 ± 0.03^C^
	Z20	0.00 ± 0.00^A^	4.09 ± 0.01^C^	4.35 ± 0.01^B^	5.32 ± 0.06^C^	5.55 ± 0.22^D^
	Z20‐HEO	0.00 ± 0.00^A^	4.05 ± 0.01^C^	4.34 ± 0.03^B^	5.31 ± 0.21^C^	5.54 ± 0.09^D^
Spraying	Z10	0.00 ± 0.00^A^	4.14 ± 0.08^B^	4.27 ± 0.00^C^	5.29 ± 0.05^D^	5.63 ± 0.06^C^
	Z10‐HEO	0.00 ± 0.00^A^	4.09 ± 0.13^C^	4.26 ± 0.01^C^	5.23 ± 0.02^D^	5.47 ± 0.06^DE^
	Z15	0.00 ± 0.00^A^	4.02 ± 0.02^C^	4.24 ± 0.01^C^	5.20 ± 0.02^DE^	5.49 ± 0.27^DE^
	Z15‐HEO	0.00 ± 0.00^A^	3.96 ± 0.01^CD^	4.21 ± 0.01^C^	5.16 ± 0.20^E^	5.45 ± 0.07^E^
	Z20	0.00 ± 0.00^A^	3.94 ± 0.00^CD^	4.14 ± 0.00^D^	5.01 ± 0.07^F^	5.39 ± 0.01^F^
	Z20‐HEO	0.00 ± 0.00^A^	3.89 ± 0.00^D^	4.10 ± 0.00^D^	5.34 ± 0.05^C^	5.25 ± 0.22^G^
Enrobing	Z10	0.00 ± 0.00^A^	4.14 ± 0.02^B^	4.24 ± 0.00^C^	5.43 ± 0.04^B^	5.54 ± 0.04^D^
	Z10‐HEO	0.00 ± 0.00^A^	4.09 ± 0.01^C^	4.21 ± 0.00^C^	5.20 ± 0.09^DE^	5.41 ± 0.09^E^
	Z15	0.00 ± 0.00^A^	3.96 ± 0.02^CD^	4.14 ± 0.01^D^	5.22 ± 0.22^D^	5.39 ± 0.01^EF^
	Z15‐HEO	0.00 ± 0.00^A^	3.94 ± 0.00^CD^	4.10 ± 0.00^D^	5.22 ± 0.09^D^	5.35 ± 0.07^F^
	Z20	0.00 ± 0.00^A^	3.89 ± 0.02^D^	4.02 ± 0.01^E^	5.03 ± 0.00^F^	5.31 ± 0.03^FG^
	Z20‐HEO	0.00 ± 0.00^A^	3.81 ± 0.03^E^	3.96 ± 0.02^F^	4.98 ± 0.20^G^	5.10 ± 0.09^H^

(B)						
Psychrotrophic bacteria (log CFU/g)						
Methods	Treatments	0	14	28	42	56
C	C	3.94 ± 0.05^A^	4.62 ± 0.02^A^	5.41 ± 0.24^A^	5.62 ± 0.28^A^	5.98 ± 0.20^A^
Brushing	Z10	3.94 ± 0.05^A^	4.57 ± 0.01^AB^	5.32 ± 0.14^B^	5.59 ± 0.27^AB^	5.54 ± 0.15^C^
	Z10‐HEO	3.94 ± 0.05^A^	4.53 ± 0.02^B^	5.25 ± 0.13^C^	5.57 ± 0.24^AB^	5.51 ± 0.13^C^
	Z15	3.94 ± 0.05^A^	4.46 ± 0.02^BC^	5.18 ± 0.01^CD^	5.52 ± 0.19^B^	5.53 ± 0.23^C^
	Z15‐HEO	3.94 ± 0.05^A^	4.44 ± 0.00^C^	5.07 ± 0.07^E^	5.43 ± 0.10^C^	5.51 ± 0.20^C^
	Z20	3.94 ± 0.05^A^	4.38 ± 0.01^D^	5.01 ± 0.12^E^	5.42 ± 0.10^C^	5.48 ± 0.15^CD^
	Z20‐HEO	3.94 ± 0.05^A^	4.36 ± 0.02^D^	5.02 ± 0.09^E^	5.34 ± 0.02^D^	5.44 ± 0.23^D^
Dipping	Z10	3.94 ± 0.05^A^	4.58 ± 0.00^AB^	5.37 ± 0.19^AB^	5.60 ± 0.28^A^	5.62 ± 0.22^B^
	Z10‐HEO	3.94 ± 0.05^A^	4.58 ± 0.00^AB^	5.29 ± 0.10^BC^	5.58 ± 0.25^AB^	5.68 ± 0.12^B^
	Z15	3.94 ± 0.05^A^	4.55 ± 0.00^B^	5.24 ± 0.08^C^	5.52 ± 0.20^B^	5.66 ± 0.19^B^
	Z15‐HEO	3.94 ± 0.05^A^	4.55 ± 0.00^B^	5.21 ± 0.09^C^	5.53 ± 0.22^B^	5.57 ± 0.16^BC^
	Z20	3.94 ± 0.05^A^	4.43 ± 0.01^C^	5.20 ± 0.13^C^	5.48 ± 0.14^BC^	5.51 ± 0.17^C^
	Z20‐HEO	3.94 ± 0.05^A^	4.41 ± 0.00^C^	5.11 ± 0.03^D^	5.43 ± 0.10^C^	5.48 ± 0.13^CD^
Spraying	Z10	3.94 ± 0.05^A^	4.33 ± 0.01^D^	5.31 ± 0.19^B^	5.54 ± 0.29^B^	5.68 ± 0.06^B^
	Z10‐HEO	3.94 ± 0.05^A^	4.30 ± 0.00^DE^	5.28 ± 0.18^C^	5.51 ± 0.28^B^	5.62 ± 0.07^B^
	Z15	3.94 ± 0.05^A^	4.34 ± 0.03^D^	5.25 ± 0.19^C^	5.48 ± 0.24^BC^	5.62 ± 0.07^B^
	Z15‐HEO	3.94 ± 0.05^A^	4.29 ± 0.01^DE^	5.22 ± 0.18^C^	5.44 ± 0.25^C^	5.54 ± 0.20^C^
	Z20	3.94 ± 0.05^A^	4.25 ± 0.00^E^	5.13 ± 0.13^D^	5.39 ± 0.22^D^	5.62 ± 0.21^B^
	Z20‐HEO	3.94 ± 0.05^A^	4.22 ± 0.03^E^	5.02 ± 0.04^E^	5.31 ± 0.14^DE^	5.48 ± 0.18^CD^
Enrobing	Z10	3.94 ± 0.05^A^	4.48 ± 0.01^C^	5.27 ± 0.16^C^	5.48 ± 0.27^BC^	5.56 ± 0.10^BC^
	Z10‐HEO	3.94 ± 0.05^A^	4.51 ± 0.03^B^	5.22 ± 0.17^C^	5.44 ± 0.26^C^	5.51 ± 0.20^C^
	Z15	3.94 ± 0.05^A^	4.44 ± 0.01^C^	5.22 ± 0.15^C^	5.36 ± 0.19^D^	5.48 ± 0.25^CD^
	Z15‐HEO	3.94 ± 0.05^A^	4.41 ± 0.04^C^	5.16 ± 0.05^D^	5.34 ± 0.17^D^	5.44 ± 0.19^D^
	Z20	3.94 ± 0.05^A^	4.40 ± 0.01^C^	5.05 ± 0.07^E^	5.30 ± 0.15^DE^	5.30 ± 0.16^E^
	Z20‐HEO	3.94 ± 0.05^A^	4.37 ± 0.00^D^	5.00 ± 0.02^EF^	5.27 ± 0.17^E^	5.30 ± 0.16^E^

(C)						
*Enterobacteriaceae* (log CFU/g)						
Methods	Treatments	0	14	28	42	56
C	C	2.82 ± 0.02^A^	2.97 ± 0.03^A^	3.32 ± 0.00^A^	3.38 ± 0.01^A^	3.56 ± 0.00^A^
Brushing	Z10	2.82 ± 0.02^A^	2.74 ± 0.04^C^	3.27 ± 0.00^B^	3.34 ± 0.00^AB^	3.43 ± 0.00^B^
	Z10‐HEO	2.83 ± 0.02^A^	2.78 ± 0.03^BC^	3.30 ± 0.00^A^	3.34 ± 0.03^AB^	3.42 ± 0.01^B^
	Z15	2.83 ± 0.02^A^	2.65 ± 0.03^D^	3.29 ± 0.00^AB^	3.29 ± 0.01^B^	3.37 ± 0.00^BC^
	Z15‐HEO	2.83 ± 0.02^A^	2.58 ± 0.14^DE^	3.30 ± 0.11^A^	3.33 ± 0.11^AB^	3.36 ± 0.02^BC^
	Z20	2.83 ± 0.02^A^	2.22 ± 0.01^G^	3.22 ± 0.00^B^	3.28 ± 0.01^B^	3.34 ± 0.05^C^
	Z20‐HEO	2.83 ± 0.02^A^	2.16 ± 0.00^GH^	3.21 ± 0.01^B^	3.27 ± 0.00^B^	3.32 ± 0.00^C^
Dipping	Z10	2.83 ± 0.02^A^	2.90 ± 0.02^AB^	3.27 ± 0.00^B^	3.31 ± 0.02^AB^	3.43 ± 0.00^B^
	Z10‐HEO	2.83 ± 0.02^A^	2.83 ± 0.01^B^	3.30 ± 0.00^A^	3.34 ± 0.00^AB^	3.40 ± 0.01^B^
	Z15	2.83 ± 0.02^A^	2.71 ± 0.00^C^	3.26 ± 0.01^B^	3.34 ± 0.00^AB^	3.38 ± 0.00^BC^
	Z15‐HEO	2.83 ± 0.02^A^	2.72 ± 0.00^C^	3.25 ± 0.00^B^	3.36 ± 0.00^A^	3.40 ± 0.01^B^
	Z20	2.83 ± 0.02^A^	2.69 ± 0.04^D^	3.21 ± 0.01^B^	3.27 ± 0.00^B^	3.38 ± 0.00^BC^
	Z20‐HEO	2.83 ± 0.02^A^	2.54 ± 0.02^E^	3.18 ± 0.00^BC^	3.26 ± 0.00^B^	3.34 ± 0.00^C^
Spraying	Z10	2.83 ± 0.02^A^	2.57 ± 0.00^DE^	2.95 ± 0.06^C^	0.00 ± 0.00^C^	0.00 ± 0.00^D^
	Z10‐HEO	2.83 ± 0.02^A^	2.52 ± 0.01^E^	2.97 ± 0.03^C^	0.00 ± 0.00^C^	0.00 ± 0.00^D^
	Z15	2.83 ± 0.02^A^	2.64 ± 0.13^D^	2.97 ± 0.03^C^	0.00 ± 0.00^C^	0.00 ± 0.00^D^
	Z15‐HEO	2.83 ± 0.02^A^	2.67 ± 0.09^D^	2.92 ± 0.03^CD^	0.00 ± 0.00^C^	0.00 ± 0.00^D^
	Z20	2.83 ± 0.02^A^	2.52 ± 0.11^DE^	2.84 ± 0.04^D^	0.00 ± 0.00^C^	0.00 ± 0.00^D^
	Z20‐HEO	2.83 ± 0.02^A^	2.44 ± 0.04^F^	2.85 ± 0.00^D^	0.00 ± 0.00^C^	0.00 ± 0.00^D^
Enrobing	Z10	2.83 ± 0.02^A^	0.00 ± 0.00^H^	0.00 ± 0.00^E^	0.00 ± 0.00^C^	0.00 ± 0.00^D^
	Z10‐HEO	2.83 ± 0.02^A^	0.00 ± 0.00^H^	0.00 ± 0.00^E^	0.00 ± 0.00^C^	0.00 ± 0.00^D^
	Z15	2.83 ± 0.02^A^	0.00 ± 0.00^H^	0.00 ± 0.00^E^	0.00 ± 0.00^C^	0.00 ± 0.00^D^
	Z15‐HEO	2.83 ± 0.02^A^	0.00 ± 0.00^H^	0.00 ± 0.00^E^	0.00 ± 0.00^C^	0.00 ± 0.00^D^
	Z20	2.83 ± 0.02^A^	0.00 ± 0.00^H^	0.00 ± 0.00^E^	0.00 ± 0.00^C^	0.00 ± 0.00^D^
	Z20‐HEO	2.83 ± 0.02^A^	0.00 ± 0.00^H^	0.00 ± 0.00^E^	0.00 ± 0.00^C^	0.00 ± 0.00^D^

(D)						
Yeast‐molds (log CFU/g)						
Methods	Treatments	0	14	28	42	56
C	C	0.00 ± 0.00^A^	1.30 ± 0.00^A^	1.93 ± 0.03^A^	2.25 ± 0.01^A^	3.08 ± 0.05^A^
Brushing	Z10	0.00 ± 0.00^A^	1.09 ± 0.01^C^	1.45 ± 0.01^D^	1.73 ± 0.03^D^	2.27 ± 0.00^FG^
	Z10‐HEO	0.00 ± 0.00^A^	1. 00 ± 0.00^C^	1.52 ± 0.01^D^	1.75 ± 0.01^D^	2.19 ± 0.02^G^
	Z15	0.00 ± 0.00^A^	1.00 ± 0.01^C^	1.50 ± 0.00^D^	1.83 ± 0.03^C^	2.18 ± 0.02^G^
	Z15‐HEO	0.00 ± 0.00^A^	0.93 ± 0.00^CD^	1.47 ± 0.00^D^	1.77 ± 0.00^D^	2.00 ± 0.01^H^
	Z20	0.00 ± 0.00^A^	0.88 ± 0.01^D^	1.40 ± 0.00^E^	1.30 ± 0.00^I^	2.00 ± 0.03^H^
	Z20‐HEO	0.00 ± 0.00^A^	0.80 ± 0. 00^DE^	1.35 ± 0.02^F^	1.30 ± 0.00^I^	1.88 ± 0.00^HI^
Dipping	Z10	0.00 ± 0.00^A^	1.22 ± 0.01^B^	1.65 ± 0.02^B^	2.17 ± 0.00^B^	2.84 ± 0.08^B^
	Z10‐HEO	0.00 ± 0.00^A^	1.19 ± 0.00^B^	1.63 ± 0.03^B^	2.14 ± 0.00^B^	2.73 ± 0.03^C^
	Z15	0.00 ± 0.00^A^	1.15 ± 0.01^BC^	1.58 ± 0.02^CD^	2.07 ± 0.00^BC^	2.67 ± 0.00^D^
	Z15‐HEO	0.00 ± 0.00^A^	1.10 ± 0.00^C^	1.47 ± 0.00^D^	2.04 ± 0.02^BC^	2.55 ± 0.02^E^
	Z20	0.00 ± 0.00^A^	1.11 ± 0.01^C^	1.52 ± 0.00^C^	1.83 ± 0.03^C^	2.33 ± 0.03^F^
	Z20‐HEO	0.00 ± 0.00^A^	1.06 ± 0.01^C^	1.47 ± 0.09^D^	1.70 ± 0.00^DE^	2.20 ± 0.01^G^
Spraying	Z10	0.00 ± 0.00^A^	0.75 ± 0.11^E^	1.60 ± 0.00^BC^	1.80 ± 0.00^C^	2.28 ± 0.00^FG^
	Z10‐HEO	0.00 ± 0.00^A^	0.73 ± 0.00^E^	1.58 ± 0.00^C^	1.80 ± 0.00^C^	2.15 ± 0.01^GH^
	Z15	0.00 ± 0.00^A^	0.65 ± 0.11^F^	1.50 ± 0.00^D^	1.65 ± 0.00^F^	2.06 ± 0.00^H^
	Z15‐HEO	0.00 ± 0.00^A^	0.55 ± 0.01^G^	0.55 ± 0.09^I^	1.58 ± 0.01^FG^	2.00 ± 0.00^H^
	Z20	0.00 ± 0.00^A^	0.00 ± 0.00^H^	0.00 ± 0.00^J^	1.00 ± 0.00^J^	1.85 ± 0.01^HI^
	Z20‐HEO	0.00 ± 0.00^A^	0.00 ± 0.00^H^	0.00 ± 0.00^J^	1.00 ± 0.00^J^	1.50 ± 0.00^K^
Enrobing	Z10	0.00 ± 0.00^A^	0.50 ± 0.00^G^	1.00 ± 0.00^G^	1.53 ± 0.03^G^	1.88 ± 0.02^HI^
	Z10‐HEO	0.00 ± 0.00^A^	0.50 ± 0.00^G^	1.00 ± 0.00^G^	1.30 ± 0.00^I^	1.80 ± 0.00^I^
	Z15	0.00 ± 0.00^A^	0.55 ± 0.01^G^	0.75 ± 0.01^H^	1.45 ± 0.01^H^	1.69 ± 0.02^J^
	Z15‐HEO	0.00 ± 0.00^A^	0.00 ± 0.00^H^	0.00 ± 0.00^J^	1.00 ± 0.00^J^	1.63 ± 0.03^JK^
	Z20	0.00 ± 0.00^A^	0.00 ± 0.00^H^	0.00 ± 0.00^J^	1.00 ± 0.00^J^	1.50 ± 0.00^K^
	Z20‐HEO	0.00 ± 0.00^A^	0.00 ± 0.00^H^	0.00 ± 0.00^J^	0.50 ± 0.00^K^	1.30 ± 0.00^L^

*Note*: Different letters within the same interval (day) (A, B, C, etc.) indicate a statistically significant difference (*p* ≤ .05).

Abbreviations: C, control; HEO, *Heracleum persicum* essential oil; Z, Zein.

Generally, the antimicrobial activities of essential oils are due to their phenolic substances, followed by their hydrophobic nature. Therefore, they can penetrate the cell walls of the microorganisms. By passing through the cell wall and gaining penetration into microorganisms, essential oils can disrupt their activities and lead to cell death (Khorshidian et al., 2018). By elongating the lag phase of bacterial growth, essential oils decrease their logarithmic growth. In other words, the accumulation of essential oils in the fatty cell membrane of bacteria causes damage to its structure and cell death. Generally, the main constituents of HEO are divided into volatile (such as aliphatic esters, carbonyls, phenyl propenes, and terpenes) and nonvolatile (such as flavonoids, furanocoumarins, tannins, and alkaloids) compounds. Aliphatic esters are the main biological constituents of HEO (Majidi & Sadati Lamardi, 2018). According to the previous studies, aliphatic esters such as hexyl butyrate, octyl 2‐methylbutyrate, octyl isobutyrate, octyl acetate, and hexyl 2‐methylbutyrate are the main antimicrobial compounds of HEO (Ghavam, 2023; Shariatifar et al., 2017).

#### 3.2.2 Psychrotrophic bacteria

Table 3 illustrates the psychrotrophic bacteria population of the treatments during 56 days of the storage time. Initial counts of them were in the range of 3.55–3.94 log CFU/g. Control samples significantly (*p* ≤ .05) showed the highest bacteria compared with the other treatments during storage time. The lowest psychrotrophic bacteria belonged to the enrobing method and Z20‐HEO, Z20, and Z15‐HEO treatments throughout the storage period, respectively. Tayebi‐Moghaddam et al. (2021) reported that the zein‐based edible films containing clove essential oil suppressed the growth of *Listeria monocytogenes* and *Escherichia coli* O157:H7 in wrapped Iranian white cheese pieces stored in 8% brine for 45 days at 4°C. Shen et al. (2022) observed that zein nanofiber membranes encapsulated with citral/Hydroxypropyl‐β‐cyclodextrin inclusion complex could impressively suppress *Listeria monocytogenes* as a psychrotrophic organism in the cold‐stored cheese samples at 4°C and 12°C. They concluded that the activated zein nanofiber membranes are an efficient solution for inhibiting *Listeria monocytogenes* contamination in cheese while preserving its color and texture features. Ünalan et al. (2013) observed strong antibacterial activities of the activated zein‐based edible film against *L. monocytogenes* in Kashar cheese.

#### 3.2.3 Enterobacteriacea

The changes in the *Enterobacteriacea* population of the studied groups are exhibited in Table 3. There was no significant difference (*p* ≥ .05) among the groups on day 0 and 2.83 CFU/g of the *Enterobacteriacea* population was found in all treatments. The enrobing and spraying methods completely inhibited the growth of bacteria on the fourteenth and 40‐second days until the end of storage time, respectively, but there was an ascending trend in the bacterial enumeration in other (coating, brushing, and control) groups by increasing the storage period. However, the uncoated cheese pieces significantly (*p* ≤ .05) showed the highest count among the others during the storage period. Pereira et al. (2020) reported that the microbial population and water loss of zein‐coated mozzarella cheese were significantly (*p* ≤ .05) lower compared with the uncoated ones during storage time. They concluded that zein‐based edible film could be introduced and used as edible packaging in the cold‐stored mozzarella cheese. Another study found the good inhibitory activity of the activated zein films against food‐borne pathogenic bacteria, including *Salmonella typhii*, *Escherchia coli*, *Proteus vulgaris*, *Enterococci faecalis*, *Pseudomonas perfringens*, *Micrococcus luteus*, and *Staphylococcus aureus* (Mushtaq et al., 2018).

#### 3.2.4 Yeast‐molds

The numeration of yeast molds in the cheese pieces is represented in Table 3. There was no yeast‐molds' colony in the studied samples on day 0. After 14 days of storage, control group showed the highest enumeration among the other groups, and the dipping, brushing, spraying, and enrobing groups were in the next ranks, respectively. Some treatments, including Z15‐HEO, Z20, and Z20‐HEO, of the spraying and enrobing methods showed the initial enumeration of yeast‐molds on day 28 of storage, which may be linked to the preservative effects of the used coating techniques. Generally, all used coating methods exhibited significant differences (*p* ≤ .05) compared with the control group all over the storage period, so enrobing was the most efficient method, and spraying, brushing, and dipping were in the next ranks. In one study, the zein‐based edible film could decrease the inoculated *Penicillium camemberti* population to approximately 2 logs on the surface of Kashar cheese during the 45‐day storage period, which is probably related to the low oxygen permeability of the used film (Küçük et al., 2020). Sadeghi Nejad et al. (2014) reported that the hydroalcoholic extract of *Heracleum persicum* fruit exhibited antifungal effects against *Candida albicans*, *Candida tropicalis*, and *Candida glabrata*. Another study by Khosravi et al. (2016) showed the antifungal activities of HEO against *Malassezia* species. According to previous research, the ethyl acetate extract of *Heracleum persicum* inhibited the growth and aflatoxin synthesis of *Aspergillus parasiticus*. It was reported that the antifungal mechanism of HEO is the destruction of cell membrane integrity, disruption in permeability, and ergosterol production of the fungal cells (Majidi & Sadati Lamardi, 2018).

### 3.3 Chemical analysis

#### 3.3.1 pH

The pH changes of the studied treatments are illustrated in Table 4. The initial pH values of the studied samples were 4.50 on day 0. A descending trend was observed in all treatments during the storage period. The enrobing, spraying, brushing, and dipping methods could significantly (*p* ≤ .05) inhibit the pH changes of the cheese pieces compared with the control during the storage time, respectively; this can be related to the antimicrobial features of them that led to a decrease in the microbial metabolites, such as lactic acid. Esparvarini et al. (2022) found a descending pattern in the pH values of the treated ultra‐filtered (UF) cheese slices during 56 days of the storage period. They reported that the uncoated cheese samples showed significantly lower pH compared with the coated ones during the storage period (*p* ≤ .05). These phenomena can be related to the decomposition of lactose into various volatile acids and lactic acid using the fermentation of lactic acid bacteria and other microorganisms. In addition, lipolysis and proteolysis reactions can produce acidic compounds in the stored cheese, such as free fatty acids and acidic amino acids (Di Pierro et al., 2011; Ramos et al., 2012; Yilmaz & Dagdemir, 2012).

**TABLE 4:** pH (A), titratable acidity (B), moisture (C), lipid (D), and lipid oxidation (E) values of the cold‐stored cheese samples.

(A)						
pH						
Methods	Treatments	0	14	28	42	56
C	C	4.50±0.00^A^	4.40±0.00^B^	3.91±0.01^D^	3.55±0.07^D^	3.05±0.01^C^
Brushing	Z10	4.50±0.00^A^	4.49±0.00^A^	4.18±0.23^C^	3.87±0.10^C^	3.77±0.03^B^
	Z10‐HEO	4.50±0.00^A^	4.45±0.00^AB^	4.11±0.08^C^	3.98±0.02^B^	3.88±0.02^AB^
	Z15	4.50±0.00^A^	4.50±0.00^A^	4.02±0.03^C^	3.84±0.08^C^	3.87±0.03^AB^
	Z15‐HEO	4.50±0.00^A^	4.50±0.00^A^	4.22±0.05^B^	4.05±0.07^B^	3.85±0.13^AB^
	Z20	4.50±0.00^A^	4.50±0.00^A^	4.06±0.07^C^	3.99±0.01^B^	3.90±0.14^A^
	Z20‐HEO	4.50±0.00^A^	4.50±0.00^A^	4.04±0.06^C^	3.74±0.07^C^	3.91±0.09^A^
Dipping	Z10	4.50±0.00^A^	4.40±0.00^B^	4.21±0.01^B^	3.94±0.05^B^	3.90±0.07^A^
	Z10‐HEO	4.50±0.00^A^	4.45±0.00^AB^	4.21±0.03^B^	3.95±0.07^B^	4.00±0.00^A^
	Z15	4.50±0.00^A^	4.50±0.00^A^	4.37±0.03^B^	3.96±0.04^B^	3.95±0.07^A^
	Z15‐HEO	4.50±0.00^A^	4.50±0.00^A^	4.34±0.06^B^	3.99±0.07^B^	3.83±0.09^AB^
	Z20	4.50±0.00^A^	4.50±0.00^A^	4.20±0.28^B^	4.00±0.16^B^	3.64±0.07^B^
	Z20‐HEO	4.50±0.00^A^	4.50±0.00^A^	4.10±0.13^C^	4.00±0.03^B^	3.92±0.10^A^
Spraying	Z10	4.50±0.00^A^	4.49±0.00^A^	4.11±0.00^C^	4.01±0.21^B^	4.00±0.00^A^
	Z10‐HEO	4.50±0.00^A^	4.49±0.00^A^	4.40±0.00^AB^	4.02±0.21^B^	3.92±0.02^A^
	Z15	4.50±0.00^A^	4.50±0.00^A^	4.11±0.00^C^	4.04±0.00^B^	3.99±0.13^A^
	Z15‐HEO	4.50±0.00^A^	4.50±0.00^A^	3.98±0.02^C^	4.07±0.02^B^	4.05±0.07^A^
	Z20	4.50±0.00^A^	4.50±0.00^A^	4.10±0.14^C^	4.06±0.02^B^	4.03±0.02^A^
	Z20‐HEO	4.50±0.00^A^	4.50±0.00^A^	4.50±0.00^A^	4.00±0.00^B^	4.02±0.03^A^
Enrobing	Z10	4.50±0.00^A^	4.49±0.00^A^	4.18±0.00^C^	4.11±0.00^B^	4.00±0.02^A^
	Z10‐HEO	4.50±0.00^A^	4.49±0.00^A^	4.10±0.00^C^	4.05±0.04^B^	3.92±0.02^A^
	Z15	4.50±0.00^A^	4.50±0.00^A^	4.25±0.00^B^	4.34±0.00^A^	3.97±0.03^A^
	Z15‐HEO	4.50±0.00^A^	4.50±0.00^A^	4.25±0.02^B^	4.37±0.02^A^	3.98±0.02^A^
	Z20	4.50±0.00^A^	4.50±0.00^A^	4.40±0.14^AB^	4.35±0.02^A^	4.15±0.08^A^
	Z20‐HEO	4.50±0.00^A^	4.50±0.00^A^	4.50±0.00^A^	4.37±0.00^A^	4.18±0.01^A^

(B)						
Titratable acidity (%)						
Methods	Treatments	0	14	28	42	56
C	C	0.67±0.00^A^	0.98±0.00^A^	1.28±0.09^A^	1.44±0.00^A^	1.81±0.06^A^
Brushing	Z10	0.67±0.00^A^	0.83±0.00^BC^	0.90±0.00^C^	1.23±0.00^C^	1.36±0.06^C^
	Z10‐HEO	0.67±0.00^A^	0.76±0.00^C^	0.85±0.06^D^	1.10±0.02^D^	1.28±0.09^D^
	Z15	0.67±0.00^A^	0.73±0.00^C^	0.78±0.06^DE^	1.05±0.15^E^	1.28±0.16^D^
	Z15‐HEO	0.67±0.00^A^	0.67±0.00^D^	0.76±0.00^E^	1.01±0.21^E^	1.28±0.09^D^
	Z20	0.67±0.00^A^	0.69±0.00^CD^	0.73±0.09^E^	1.01±0.15^E^	1.14±0.09^E^
	Z20‐HEO	0.67±0.00^A^	0.67±0.00^D^	0.69±0.09^F^	0.78±0.16^GH^	1.07±0.06^F^
Dipping	Z10	0.67±0.00^A^	0.95±0.00^AB^	1.10±0.00^B^	1.33±0.00^B^	1.46±0.06^B^
	Z10‐HEO	0.67±0.00^A^	0.90±0.00^B^	1.00±0.06^B^	1.25±0.02^C^	1.38±0.09^C^
	Z15	0.67±0.00^A^	0.88±0.00^B^	0.98±0.06^BC^	1.15±0.15^D^	1.28±0.16^D^
	Z15‐HEO	0.67±0.00^A^	0.80±0.00^BC^	0.96±0.00^BC^	1.11±0.21^D^	1.23±0.09^D^
	Z20	0.67±0.00^A^	0.78±0.00^C^	0.83±0.09^D^	1.01±0.15^E^	1.14±0.09^E^
	Z20‐HEO	0.67±0.00^A^	0.73±0.00^C^	0.60±0.09^F^	0.98±0.16^EF^	1.07±0.06^F^
Spraying	Z10	0.67±0.00^A^	0.83±0.00^BC^	1.10±0.00^B^	1.25±0.00^C^	1.35±0.00^C^
	Z10‐HEO	0.67±0.00^A^	0.75±0.02^C^	1.07±0.06^B^	1.25±0.02^C^	1.30±0.11^C^
	Z15	0.67±0.00^A^	0.69±0.02^AB^	0.97±0.06^BC^	1.15±0.00^D^	1.25±0.00^D^
	Z15‐HEO	0.67±0.00^A^	0.67±0.00^D^	0.95±0.02^C^	1.05±0.00^E^	1.32±0.10^C^
	Z20	0.67±0.00^A^	0.67±0.00^D^	0.89±0.03^D^	0.93±0.03^F^	1.23±0.16^D^
	Z20‐HEO	0.67±0.00^A^	0.67±0.00^D^	0.76±0.00^E^	0.82±0.03^G^	1.17±0.00^E^
Enrobing	Z10	0.67±0.00^A^	0.76±0.00^C^	1.05±0.03^B^	1.15±0.00^D^	1.30±0.09^C^
	Z10‐HEO	0.67±0.00^A^	0.70±0.00^CD^	1.03±0.00^B^	1.18±0.09^D^	1.30±0.06^C^
	Z15	0.67±0.00^A^	0.69±0.00^CD^	0.90±0.02^C^	1.11±0.00^D^	1.26±0.15^D^
	Z15‐HEO	0.67±0.00^A^	0.67±0.00^D^	0.78±0.00^DE^	1.02±0.02^E^	1.13±0.31^AB^
	Z20	0.67±0.00^A^	0.67±0.00^D^	0.76±0.06^E^	0.88±0.09^G^	1.10±0.09^EF^
	Z20‐HEO	0.67±0.00^A^	0.67±0.00^D^	0.69±0.02^F^	0.77±0.06^GH^	0.86±0.15^G^

(C)						
Moisture (%)						
Methods	Treatments	0	14	28	42	56
C	C	65.00±0.00^A^	55.00±0.23^D^	52.99±0.24^C^	48.66±0.30^C^	40.50±0.53^D^
Brushing	Z10	65.00±0.00^A^	63.83±0.24^A^	62.49±0.23^B^	60.83±0.24^B^	59.16±0.23^A^
	Z10‐HEO	65.00±0.00^A^	64.16±0.23^A^	63.16±0.23^AB^	61.16±0.23^A^	59.83±0.17^A^
	Z15	65.00±0.00^A^	64.16±0.23^A^	62.33±0.16^B^	60.33±0.41^B^	58.00±0.00^B^
	Z15‐HEO	65.00±0.00^A^	63.83±0.24^A^	63.00±0.00^AB^	61.16±0.23^A^	59.99±0.24^A^
	Z20	65.00±0.00^A^	64.33±0.26^A^	63.99±0.17^A^	62.16±0.23^A^	60.16±0.18^A^
	Z20‐HEO	65.00±0.00^A^	64.66±0.27^A^	63.33±0.24^A^	60.49±0.164^B^	61.00±0.00^A^
Dipping	Z10	65.00±0.00^A^	60.66±0.18^C^	61.16±0.23^BC^	60.49±0.23^B^	54.16±0.42^C^
	Z10‐HEO	65.00±0.00^A^	62.16±0.23^B^	61.83±0.24^BC^	61.33±0.26^A^	54.16±0.13^C^
	Z15	65.00±0.00^A^	62.49±0.23^B^	61.33±0.46^BC^	60.16±0.18^B^	58.83±0.58^B^
	Z15‐HEO	65.00±0.00^A^	62.00±0.00^B^	61.16±0.23^BC^	60.83±0.24^B^	60.33±0.24^A^
	Z20	65.00±0.00^A^	62.33±0.16^B^	62.16±0.23^B^	61.16±0.70^A^	60.00±0.41^A^
	Z20‐HEO	65.00±0.00^A^	62.49±0.23^B^	62.00±0.00^B^	61.99±0.47^A^	59.33±0.46^A^
Spraying	Z10	65.00±0.00^A^	63.99±0.17^A^	62.49±0.23^B^	61.99±0.47^A^	58.50±0.70^B^
	Z10‐HEO	65.00±0.00^A^	64.16±0.20^A^	61.99±0.14^BC^	62.50±0.70^A^	60.50±0.70^A^
	Z15	65.00±0.00^A^	62.66±0.18^B^	62.99±0.24^B^	59.16±0.23^B^	58.66±0.47^B^
	Z15‐HEO	65.00±0.00^A^	62.99±0.14^B^	62.50±0.10^B^	62.16±0.70^A^	61.83±0.24^A^
	Z20	65.00±0.00^A^	64.33±0.24^A^	62.50±0.20^B^	60.50±0.70^B^	60.66±0.44^A^
	Z20‐HEO	65.00±0.00^A^	65.00±0.00^A^	64.16±0.10^A^	62.33±0.46^A^	59.99±0.14^A^
Enrobing	Z10	65.00±0.00^A^	63.99±0.17^A^	62.49±0.23^B^	60.33±0.18^B^	59.83±0.70^A^
	Z10‐HEO	65.00±0.00^A^	64.16±0.70^A^	63.99±0.44^A^	60.66±0.35^B^	60.66±0.47^A^
	Z15	65.00±0.00^A^	63.66±0.18^A^	64.99±0.24^A^	62.16±0.42^A^	59.50±0.70^A^
	Z15‐HEO	65.00±0.00^A^	63.99±0.34^A^	64.50±0.20^A^	61.50±0.53^A^	59.49±0.23^A^
	Z20	65.00±0.00^A^	64.33±0.14^A^	64.50±0.10^A^	62.16±0.00^A^	60.49±0.00^A^
	Z20‐HEO	65.00±0.00^A^	65.00±0.00^A^	64.16±0.10^A^	61.83±0.65^A^	60.99±0.24^A^

(D)						
Lipid (%)						
Methods	Treatments	0	14	28	42	56
C	C	4.90±0.00^A^	4.30±0.00^F^	3.80±0.00^F^	3.00±0.35^D^	2.10±0.00^D^
Brushing	Z10	4.90±0.00^A^	4.50±0.00^D^	4.00±0.00^E^	3.70±0.28^C^	3.10±0.14^C^
	Z10‐HEO	4.90±0.00^A^	4.50±0.00^D^	4.00±0.00^E^	3.90±0.00^C^	3.50±0.00^BC^
	Z15	4.90±0.0^A^	4.65±0.14^C^	4.20±0.00^E^	3.50±0.00^CD^	3.75±0.15^B^
	Z15‐HEO	4.90±0.00^A^	4.60±0.14^C^	4.00±0.00^E^	3.75±0.35^C^	3.75±0.07^B^
	Z20	4.90±0.00^A^	4.80±0.28^AB^	4.50±0.00^D^	4.00±0.00^BC^	3.95±0.07^B^
	Z20‐HEO	4.90±0.00^A^	4.90±0.00^A^	4.50±0.00^D^	4.00±0.00^BC^	3.95±0.15^B^
Dipping	Z10	4.90±0.0^A^	4.45±0.07^E^	4.00±0.00^E^	3.50±0.00^CD^	3.00±0.00^C^
	Z10‐HEO	4.90±0.00^A^	4.50±0.00^D^	4.10±0.14^E^	3.70±0.28^C^	3.10±0.14^C^
	Z15	4.90±0.00^A^	4.50±0.00^D^	4.10±0.14^E^	3.35±0.21^CD^	3.25±0.35^C^
	Z15‐HEO	4.90±0.00^A^	4.40±0.00^E^	4.10±0.14^E^	3.50±0.00^CD^	3.10±0.14^C^
	Z20	4.90±0.00^A^	4.50±0.00^D^	4.25±0.35^DE^	3.70±0.28^C^	3.50±0.00^BC^
	Z20‐HEO	4.90±0.00^A^	4.50±0.00^D^	4.50±0.00^D^	3.95±0.07^C^	3.70±0.28^B^
Spraying	Z10	4.90±0.00^A^	4.60±0.00^C^	4.50±0.00^D^	3.50±0.00^CD^	3.10±0.14^C^
	Z10‐HEO	4.90±0.00^A^	4.60±0.21^C^	4.50±0.00^D^	3.75±0.35^C^	3.50±0.00^BC^
	Z15	4.90±0.00^A^	4.65±0.00^C^	4.60±0.21^C^	3.75±0.35^C^	4.00±0.00^AB^
	Z15‐HEO	4.90±0.00^A^	4.60±0.21^C^	4.60±0.14^C^	4.10±0.14^BC^	3.95±0.35^B^
	Z20	4.90±0.00^A^	4.70±0.21^BC^	4.65±0.49^BC^	4.10±0.14^BC^	4.00±0.00^AB^
	Z20‐HEO	4.90±0.00^A^	4.70±0.00^BC^	4.67±0.21^BC^	4.35±0.21^B^	4.10±0.14^AB^
Enrobing	Z10	4.90±0.00^A^	4.75±0.21^B^	4.65±0.21^BC^	4.35±0.00^B^	4.15±0.35^AB^
	Z10‐HEO	4.90±0.00^A^	4.70±0.28^B^	4.67±0.00^BC^	4.35±0.21^B^	4.25±0.28^A^
	Z15	4.90±0.00^A^	4.85±0.00^AB^	4.75±0.35^B^	4.50±0.00^A^	4.10±0.14^AB^
	Z15‐HEO	4.90±0.00^A^	4.90±0.14^A^	4.75±0.14^B^	4.50±0.14^A^	3.95±0.00^B^
	Z20	4.90±0.00^A^	4.90±0.28^A^	4.85±0.21^A^	4.50±0.00^A^	4.35±0.21^A^
	Z20‐HEO	4.90±0.00^A^	4.90±0.00^A^	4.90±0.00^A^	4.50±0.00^A^	4.30±0.14^A^

(E)						
TBARS value (mg MDA/kg sample)						
Methods	Treatments	0	14	28	42	56
C	C	0.06±0.08^A^	0.24±0.01 ^A^	0.57±0.03^A^	0.89±0.00^A^	1.28±0.03^A^
Brushing	Z10	0.06±0.08^A^	0.18±0.09^B^	0.49±0.12^B^	0.65±0.01^C^	1.04±0.00^B^
	Z10‐HEO	0.06±0.08^A^	0.17±0.00^B^	0.50±0.00^B^	0.61±0.01^C^	0.97±0.01^B^
	Z15	0.06±0.03^A^	0.13±0.01^C^	0.45±0.11^BC^	0.64±0.01^C^	0.88±0.12^C^
	Z15‐HEO	0.06±0.07^A^	0.14±0.02^C^	0.41±0.12^C^	0.58±0.04^D^	0.82±0.02^CD^
	Z20	0.06±0.06^A^	0.12±0.02^D^	0.43±0.02^BC^	0.60±0.01^CD^	0.77±0.02^D^
	Z20‐HEO	0.06±0.08^A^	0.10±0.07^E^	0.41±0.13^C^	0.57±0.01^D^	0.68±0.02^E^
Dipping	Z10	0.05±0.07^A^	0.19±0.00^B^	0.55±0.12^A^	0.71±0.00^B^	1.19±0.01^AB^
	Z10‐HEO	0.06±0.04^A^	0.18±0.03^B^	0.51±0.10^A^	0.69±0.00^B^	1.10±0.00^AB^
	Z15	0.06±0.06^A^	0.16±0.06^BC^	0.46±0.11^BC^	0.65±0.01^C^	1.06±0.01^B^
	Z15‐HEO	0.05±0.00^A^	0.14±0.02^C^	0.45±0.11^BC^	0.62±0.00^C^	1.09±0.00^B^
	Z20	0.06±0.00^A^	0.16±0.04^BC^	0.50±0.02^B^	0.60±0.00^C^	1.01±0.04^B^
	Z20‐HEO	0.06±0.09^A^	0.17±0.01^B^	0.43±0.13^BC^	0.58±0.00^D^	0.88±0.00^C^
Spraying	Z10	0.06±0.07^A^	0.18±0.07^B^	0.45±0.05^BC^	0.63±0.01^C^	0.84±0.01^C^
	Z10‐HEO	0.06±0.06^A^	0.19±0.08^B^	0.44±0.06^BC^	0.58±0.06^D^	0.82±0.02^CD^
	Z15	0.05±0.02^A^	0.15±0.06^C^	0.39±0.05^C^	0.62±0.01^C^	0.95±0.08^B^
	Z15‐HEO	0.05±0.04^A^	0.17±0.02^B^	0.40±0.01^C^	0.52±0.01^DE^	0.78±0.00^D^
	Z20	0.06±0.04^A^	0.12±0.06^D^	0.35±0.07^D^	0.48±0.00^E^	0.66±0.00^E^
	Z20‐HEO	0.05±0.08^A^	0.14±0.05^C^	0.29±0.02^E^	0.40±0.00^EF^	0.54±0.00^F^
Enrobing	Z10	0.06±0.02^A^	0.19±0.01^B^	0.41±0.00^C^	0.57±0.01^D^	0.73±0.02^D^
	Z10‐HEO	0.06±0.09^A^	0.16±0.00^BC^	0.41±0.08^C^	0.59±0.02^D^	0.75±0.07^D^
	Z15	0.05±0.00^A^	0.13±0.01^C^	0.42±0.03^C^	0.55±0.08^D^	0.72±0.40^D^
	Z15‐HEO	0.05±0.00^A^	0.11±0.03^DE^	0.37±0.06^D^	0.47±0.07^E^	0.60±0.11^EF^
	Z20	0.04±0.00^A^	0.09±0.00^E^	0.28±0.04^E^	0.41±0.10^EF^	0.56±0.05^F^
	Z20‐HEO	0.04±0.05^A^	0.07±0.02^F^	0.20±0.08^F^	0.37±0.16^F^	0.50±0.10^G^

*Note*: Different letters within the same interval (day) (A, B, C, etc.) indicate a statistically significant difference (*p* ≤ .05).

Abbreviations: C, control; HEO, *Heracleum persicum* essential oil; Z, Zein.

#### 3.3.2 Titratable acidity

The trend of titratable acidity of the studied treatments is represented in Table 4B. The amount of 0.67% was calculated as an initial TA for all of the treatments. An increasing trend was found in the TA of all groups throughout the storage time, so uncoated samples showed the highest TA compared with the other groups over time. The lowest TA was found in the enrobing method in the Z20‐HEO treatment in all intervals, which can be due to the preservative effects of this treatment on the chemical changes of the samples. In other words, En‐Z20‐HEO treatment significantly (*p* ≤ .05) lowered the microbial, chemical, and physical changes of the cheese pieces throughout the refrigerated storage that may be linked to the formation of the efficient preservative layer around the samples. Esparvarini et al. (2022) found the same pattern in our results in the TA of the treated UF cheese slices during 56 days of storage. They concluded that the starch‐gelatin edible coating containing cumin essential oil could significantly (*p* ≤ .05) prevent the TA changes of the cheese samples compared with the uncoated ones, which can be related to the complete uniform coating around the cheese slices.

#### 3.3.3 Protein and ash

According to the obtained results, there was no change in the protein (12%) and ash (6%) values of all studied treatments during the 56‐day storage period. In agreement with our findings, Pena‐Serna et al. (2016) observed no significant differences (*p* ≥ .05) in protein and ash values of the zein‐coated Minas Padrao cheese during the cold storage period. Another study reported no changes in the protein and ash values of the gelatin/starch‐coated UF cheese slices throughout the storage period (Esparvarini et al., 2022).

#### 3.3.4 Moisture

The changes in moisture percent of the studied samples are presented in Table 4B. The initial moisture percent of the samples was 65% on day 0. All of the coated cheese pieces significantly (*p* ≤ .05) inhibited moisture loss compared with the uncoated ones (control samples) all over the storage period. The coated pieces using the enrobing method were the wettest samples among the other methods at the end of the storage period. Similar patterns of moisture percent to our results were reported in other kinds of coated cheese (gelatin/starch coating in UF cheese (Esparvarini et al., 2022), zein coating in Minas Padrao cheese (Pena‐Serna et al., 2016), and whey protein isolate coating in Saloio cheese (Ramos et al., 2012)) that can be correlated to the impenetrability of the used coatings against water vapor. Moisture percent of the coated samples is dependent on the integrity, microstructure, and uniformity of the used coating because moisture could be lost from pores and cracks in the used coating. The more uniform and complete the coating, the lower the moisture loss in the coated samples (Wang et al., 2023).

#### 3.3.5 Lipid

Table 4C displays the trend of the lipid percent of the studied treatments during the storage time. The lipid content of all samples was 4.90% on day 0 and decreased by increasing the storage time. The lowest lipid content belonged to the control samples, and dipping, brushing, spraying, and enrobing treatments were in the next ranks, respectively. In other words, the enrobing technique was the best method for preserving the fat loss of the stored cheese pieces. Also, the higher zein concentration significantly (*p* ≤ .05) increased the preservative effects of the used coating methods against fat loss of the samples during the storage period. Lipid oxidation and lipolysis can decrease the lipid content of the stored cheese. By decreasing these phenomena, the lipid content of the cheese is preserved. Esparvarini et al. (2022) observed a similar trend to our results in the lipid content of the coated UF cheese during storage time. They reported that the used coating in UF cheese samples could significantly (*p* ≤ .05) decrease lipid loss compared with the uncoated slices. Pena‐Serna et al. (2016) obtained the same results about the preservative effects of zein composite coating on the lipid content of Minas Padrao cheese cubes during the storage period.

#### 3.3.6 Lipid oxidation

The TBARS values of the studied cheese pieces are represented in Table 4D. Initial TBARS values of the studied samples were in the range of 0.04–0.06 mg MDA/kg sample, and no significant difference was observed among them. The highest TBARS value was found in the uncoated pieces throughout the storage time. Zein concentration, HEO presence, and the kind of coating method significantly (*p* ≤ .05) affected the TBARS values of the treatments during the storage period, so Z20‐HEO treatment in the enrobing method showed the lowest TBARS value, and Z20, Z15‐HEO, Z15, Z10‐HEO, and Z10 were in the next ranks, respectively. This pattern was observed in other kinds of coating methods as well. Since free zein biopolymer does not have antioxidant properties, it seems that the delaying of lipid oxidation of the studied samples is due to the zein inhibitory effects of oxygen, light, and microorganism penetration into the samples and, consequently, the delaying of bacterial growth and chemical reactions (lipid oxidation) of the coated cheese. However, adding essential oils such as HEO to the coatings can cause their antioxidant properties, which is due to the antioxidant compounds of HEO that have already been proven by antioxidant tests in many previous studies (Abdollahi et al., 2022; Esparvarini et al., 2022; Ünalan et al., 2013). In agreement with our results, Esparvarini et al. (2022) observed an upward trend in the lipid oxidation of UF cheese samples during refrigerated storage time. They reported that the activated starch/gelatin‐coated cheese slices showed the lowest lipid oxidation compared with the uncoated ones. They concluded that the enrichment of the gelatin/starch coating with cumin essential oil showed the highest antioxidant activity in cheese slices over the storage period. Another study showed that the activated zein composite film produced by antioxidant agents could significantly (*p* ≤ .05) decrease lipid oxidation in coated Kashar cheese in comparison with the uncoated samples during 35 days of cold storage (Ünalan et al., 2013).

### 3.4 Physical analysis

#### 3.4.1 Color

**FIGURE 1:**
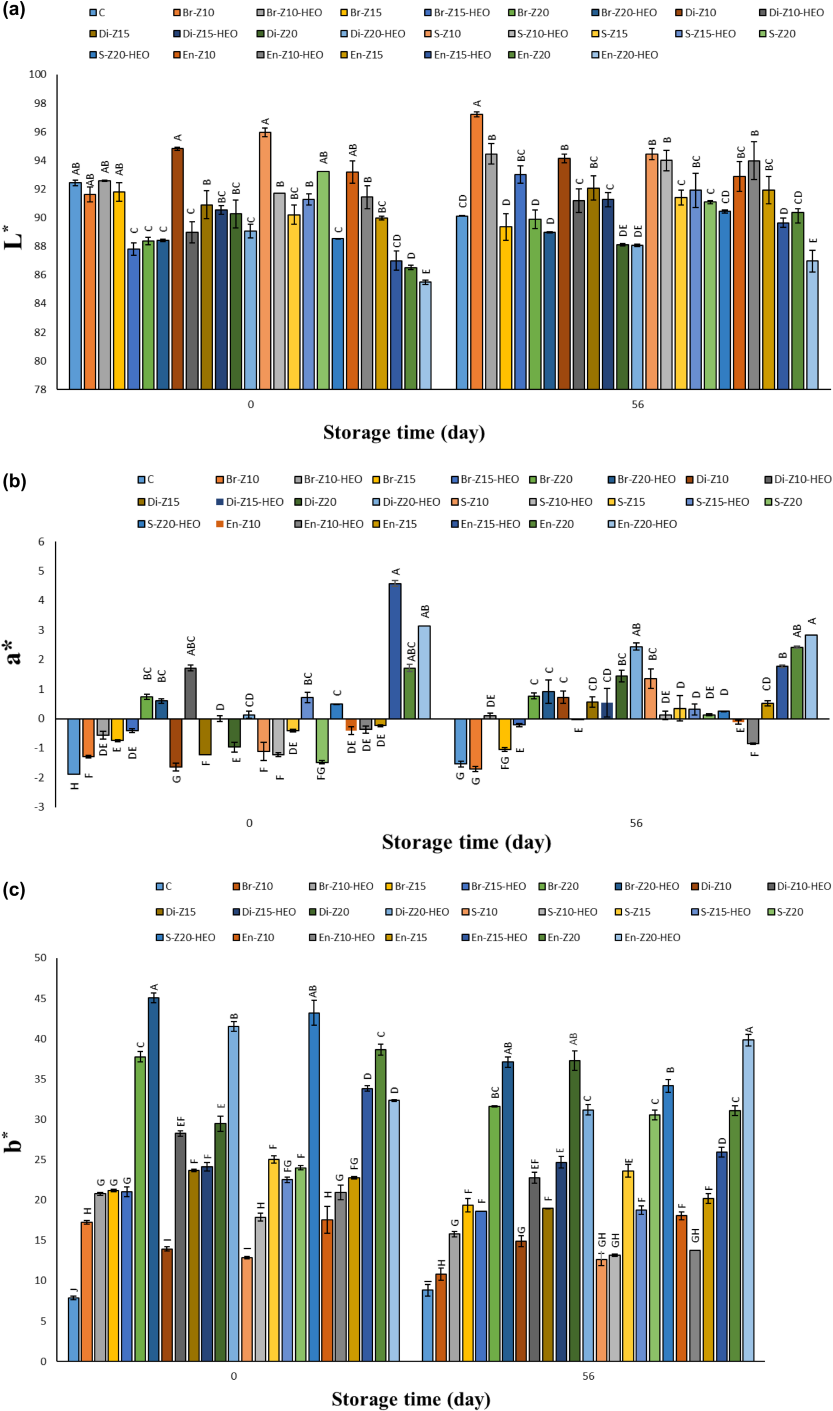
(a–c) Color characteristics of the cheese pieces during the storage period. Different letters within the same interval (day) (A, B, C, etc.) indicate a statistically significant difference (*p* ≤ .05). C: control, Br: Brushing method, Di: Dipping method, S: Spraying method, En: Enrobing method, Z: Zein; HEO: *Heracleum persicum* essential oil.

The colorimetric changes of the samples are illustrated in Image 1A‐C. By increasing the storage period, L* factor [brightness (+)/darkness (−)] significantly (*p* ≤ .05) decreased in the uncoated samples; but, in the coated pieces, especially in the higher concentrations of zein and HEO presence, no significant differences were observed in L* factor that can be related to the preservative effects of the used coating during the storage period (Figure 1). In other words, zein biopolymer could preserve the brightness of the cheese pieces during the 56‐days storage period in a dose‐dependent manner. By increasing the storage period, a* factor [red (+)/green (−)] in the treatments containing the higher concentration of zein coating increased, which can be due to the more absorption and stabilization of the coating in the samples over time (Figure 1B). Zein‐based edible coating significantly (*p* ≤ .05) caused the higher b* factor [yellow (+)/blue (−)] of the samples, which is due to the yellowish nature of the zein polymer. The lowest and highest yellowness were related to the control and Z20% groups, respectively. Pena‐Serna et al. (2016) observed a yellowish color change in Minas Padrao cheese slices after dipping them in zein solution that can be related to the natural yellow color of zein polymer. They reported that an opaque appearance was created on the surface of the uncoated cheese slices by increasing the storage period, which was in agreement with the present study. Dehydration, lipolysis, and fat oxidation phenomena lowered the initial brightness of the cheese during storage time. But the edible coatings can significantly (*p* ≤ .05) inhibit these reactions due to their lower light and gas (such as oxygen and water vapor) permeability. Also, enriching the used coatings with antioxidant and antimicrobial agents can significantly (*p* ≤ .05) improve these efficiencies (Cerqueira et al., 2010; Esparvarini et al., 2022; Ramos et al., 2012).

#### 3.4.2 Hardness

**FIGURE 2:**
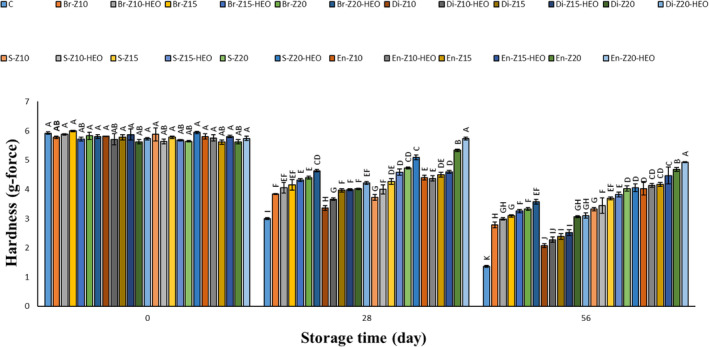
Hardness value of the cheese pieces during storage period. Different letters within the same interval (day) (A, B, C, etc.) indicate a statistically significant difference (*p* ≤ .05). C: control, Br: Brushing method, Di: Dipping method, S: Spraying method, En: Enrobing method, Z: Zein, HEO: *Heracleum persicum* essential oil.

The changes in the sample hardness are displayed in Figure 2. No significant difference was found in the initial hardness of all samples on day 0. A descending trend was observed in all of the cheese pieces as storage period increased. According to the obtained findings, the lowest hardness belonged to the uncoated samples, and the highest hardness was related to the EN‐Z20‐HEO, EN‐Z20, and EN‐Z15‐HEO treatments, respectively. It seems that the zein coating using enrobing, spraying, brushing, and dipping techniques could significantly (*p* ≤ .05) preserve the stability and hardness of the cheese texture by decreasing their microbial, chemical, and physical changes over time, respectively. Also, the zein solution obtained by the enrobing method was well dispersed and could be completely adsorbed onto the cheese surface, achieving an all‐round coating. Wang et al. (2023) investigated the effects of dipping, brushing, spraying, and electrostatic spraying coating methods by chitosan on the shelf‐life of the mango fruits during refrigerated storage. They reported that the coated mangos with the dipping method showed the highest hardness among the other treatments during the storage period, and electrostatic spraying, spraying, and brushing were in the next ranks, respectively. Another study found an ascending trend of hardness in the texture of the coted Mozzarella cheese by dipping, electrostatic spraying, spraying, and brushing methods during the storage time. They concluded that water loss caused the hardness increase of the studied cheese so that the uncoated samples showed the highest hardness while dipping, enrobing, spraying, and electrostatic spraying were in the next categories, which can be attributed to the water retention ability of the used coatings (Zhong et al., 2014). These contradictions with our findings can probably be related to the difference in the duration of the storage period, so short‐term storage causes the harder texture of cheese samples due to the moisture loss, but long‐term storage leads to the softer texture due to the chemical and microbial changes in them.

#### 3.4.3 Images

**FIGURE 3:**
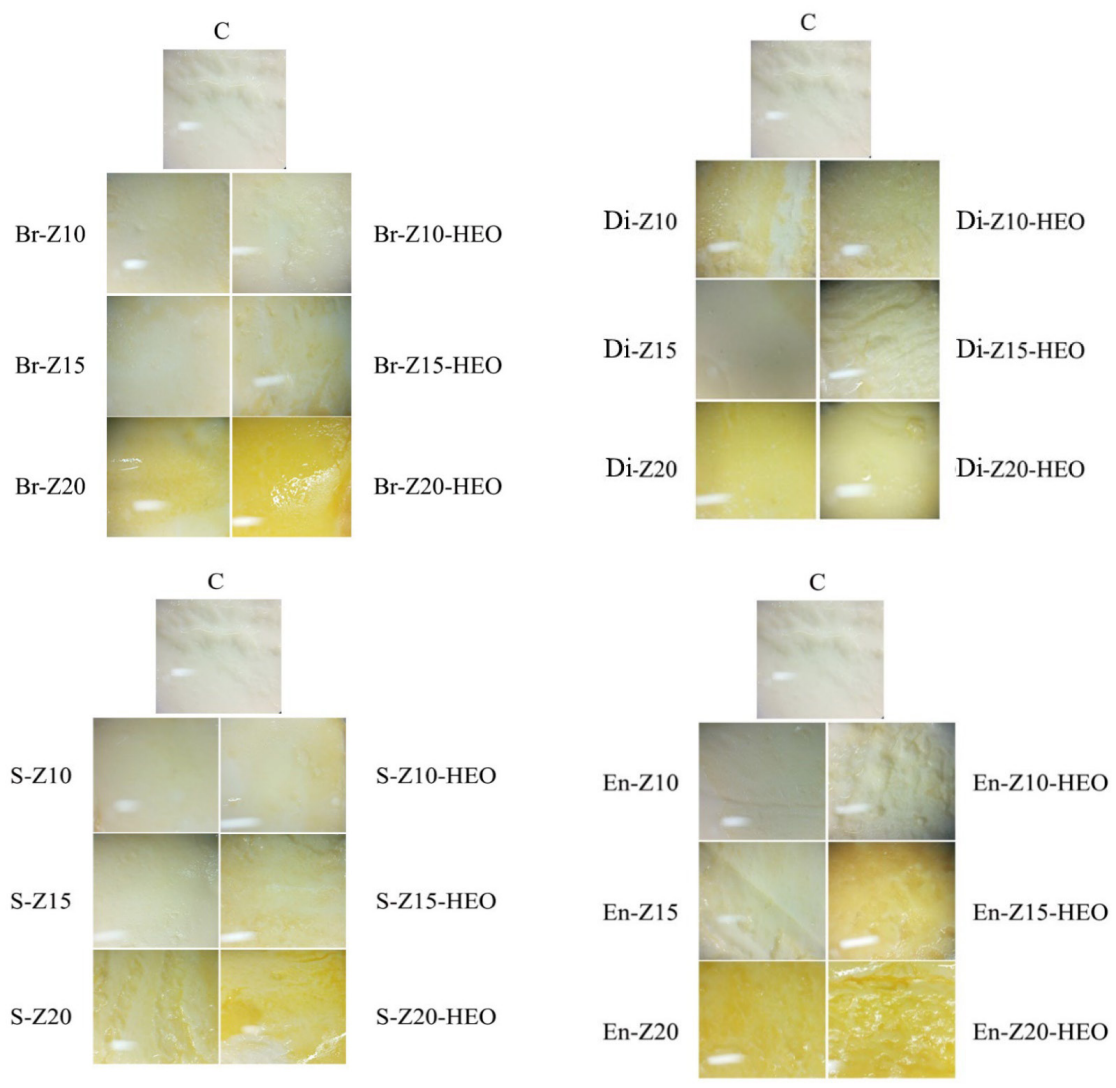
The surfaces of the cheese pieces immediately after coating. C: control, Br: Brushing method, Di: Dipping method, S: Spraying method, En: Enrobing method, Z: Zein, HEO: *Heracleum persicum* essential oil.

**FIGURE 4:**
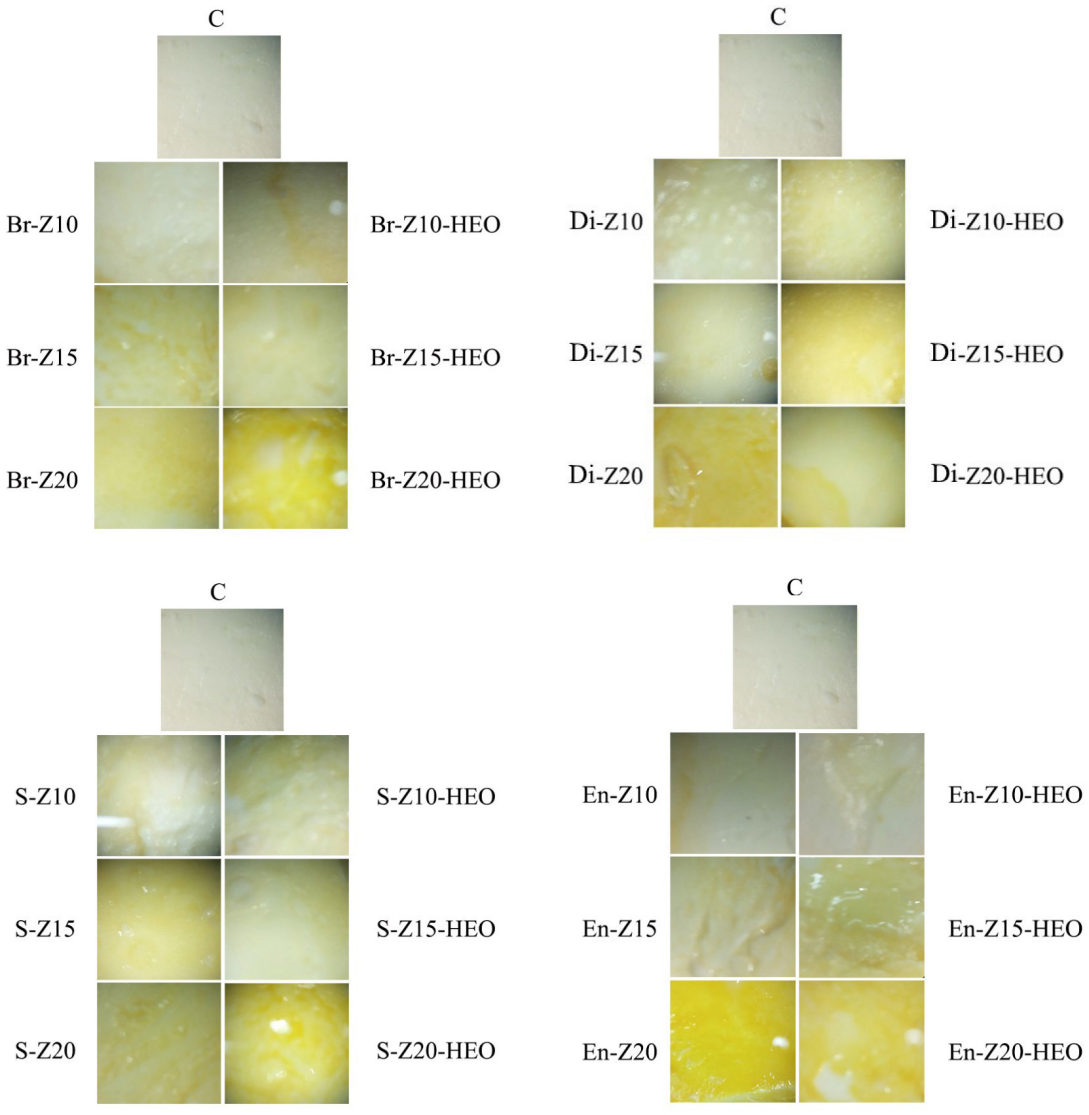
The surfaces of the cheese pieces after 56 days of storage period. C: control, Br: Brushing method, Di: Dipping method, S: Spraying method, En: Enrobing method, Z: Zein, HEO: *Heracleum persicum* essential oil.

Figure 3 illustrates the appearance of the treated cheese pieces under a stereoscope microscope on days 0 and 56. According to the obtained images, the enrobing method in zein 20% (EN‐Z20 and EN‐Z20‐HEO treatments) created a homogenous, stable, complete layer on the surface of the cheese pieces that fully covered the samples, and according to the microbial, chemical, and physical analyses, it led to the highest efficiency in the shelf‐life enhancement of the cheese pieces among the treatments during the storage period. The coating formed by the enrobing method was thick, complete, and uniform, which could significantly (*p* ≤ .05) decrease the physicochemical changes of the cheese pieces compared with the other treatments during the storage period. The spraying method created a rougher layer on the surface of the cheese pieces. During the spraying method, small droplets were quickly thrown one after another on the cheese surface, which led to the formation of droplet clusters at different points on the cheese surface, which was followed by its rough appearance. Zhong et al. (2014) observed a similar view to our finding in the coated Mozzarella cheese pieces by the spraying method. An uneven, jagged layer was formed on the surface of the cheese samples by brush traces in the brushing method, which led to the formation of cracks and pores, resulting in the quality loss of the stored cheese pieces in comparison with the enrobing and spraying methods. The dipping method formed a very thin, uniform layer on the surface of the samples, which was quickly absorbed and turned into an incomplete and uneven layer. In one study, chitosan‐based edible coating created the same appearance as our results on the coated mango surfaces by brushing and dipping methods (Wang et al., 2023) (Figure 4).

### 3.5 Sensory analysis

Changes in the sensory characteristics (taste, odor, and overall acceptability) of the cheese slices during the storage period are presented in Table 5A–C. There were no significant differences among all groups in terms of sensory characteristics at day 0. A descending pattern was observed in the sensory features of all treatments after 28 days of storage. Based on the findings of the sensory analyses, the uncoated samples (control) received the lowest scores from the panelists, while the highest scores belonged to the enrobing, spraying, brushing, and dipping methods, respectively. In agreement with the microbial and chemical analyses, the enrobing method was the most efficient technique for preserving the sensory features of the cheese slices during storage. This can be related to the inhibitory effects of the enrobing method on the microbial and chemical changes of the studied samples over the storage time. Furthermore, the higher concentrations of zein could significantly (*p* ≤ .05) receive higher sensory scores as compared to the lower concentrations. This can be due to the good preservation of zein 20% and 15% in cheese samples. Also, the combined treatments containing HEO showed more pleasant sensory features (*p* ≤ .05) compared to the others after 56 days of storage, which can be correlated to the release of the desirable taste and aroma of HEO in samples. The researchers also reported similar sensory properties to our results in reducing sensory scores in uncoated specimens. Yilmaz and Dagdemir (2012) showed that the highest overall acceptability score belonged to coated Kashar cheese with beeswax compared to uncoated or vacuum‐packed samples during 120 days of storage. Esparvarini et al. (2022) reported that a composite coating of gelatin‐starch (GS) containing cucumber peel extract (CPE) and cumin essential oil (CEO) could improve the microbial, physical, chemical, and sensory features (taste, odor, texture, and overall acceptability) of UF cheese during the 56‐day storage period. They concluded that sensory evaluation of the preparations showed that the GS coating containing CPE and CEO significantly (*p* ≤ .05) had pleasant effects on the sensory features of the cheese samples during storage time, which could be due to the preservative effects of the used coating and the pleasant taste and aroma of CPE and CEO. Abdollahi et al. (2022) observed that the Aloe vera gel (AVG) coating containing *Heracleum persicum* essential oil (HEO) not only did not have inappropriate effects on the organoleptic features of the orange slices but also gave a palatable and pleasant feeling to the panelists. They reported that HEO could significantly (*p* < .05) prevent a severe descending trend in the overall acceptability of the samples as compared to the others during storage. In other words, HEO inhibited the overall acceptability loss of the samples during the 20‐day storage period.

**TABLE 5:** Taste (A), odor (B), and overall acceptability (C) scores of the cold stored cheese samples.

(A)				
Taste				
Methods	Treatments	0	28	56
C	C	5.00±0.00^A^	3.6±0.45^E^	—
Brushing	Z10	5.00±0.00^A^	4.5±0.45^C^	3.5±0.84^E^
	Z10‐HEO	5.00±0.00^A^	4.7±0.45^B^	3.8±0.45^DE^
	Z15	5.00±0.00^A^	4.5±0.45^C^	3.8±0.71^DE^
	Z15‐HEO	5.00±0.00^A^	4.6±0.55^BC^	4.00±0.82^D^
	Z20	5.00±0.00^A^	4.5±0.55^C^	4.00±0.55^D^
	Z20‐HEO	5.00±0.00^A^	4.7±0.55^B^	4.2±0.55^CD^
Dipping	Z10	5.00±0.00^A^	4.2±0.45^D^	3.00±0.45^F^
	Z10‐HEO	5.00±0.00^A^	4.5±0.45^C^	3.2±0.82^EF^
	Z15	5.00±0.00^A^	4.5±0.82^C^	3.5±0.71^E^
	Z15‐HEO	5.00±0.00^A^	4.5±0.71^C^	3.5±0.55^E^
	Z20	5.00±0.00^A^	4.6±0.82^BC^	3.00±0.45^F^
	Z20‐HEO	5.00±0.00^A^	4.6±0.83^BC^	3.8±0.71^DE^
Spraying	Z10	5.00±0.00^A^	4.5±0.55^C^	3.6±0.82^E^
	Z10‐HEO	5.00±0.00^A^	4.7±0.56^B^	3.8±0.82^DE^
	Z15	5.00±0.00^A^	4.6±0.55^BC^	3.8±0.45^DE^
	Z15‐HEO	5.00±0.00^A^	4.7±0.71^B^	4.00±0.55^D^
	Z20	5.00±0.00^A^	4.7±0.84^B^	4.3±0.45^CD^
	Z20‐HEO	5.00±0.00^A^	5.00±0.82^A^	4.5±0.72^C^
Enrobing	Z10	5.00±0.00^A^	4.6±0.45^BC^	3.5±0.82^E^
	Z10‐HEO	5.00±0.00^A^	4.5±0.55^C^	3.8±0.55^DE^
	Z15	5.00±0.00^A^	4.6±0.45^BC^	4.00±0.84^D^
	Z15‐HEO	5.00±0.00^A^	5.00±0.71^A^	4.5±0.45^C^
	Z20	5.00±0.00^A^	5.00±0.82^A^	4.8±0.55^B^
	Z20‐HEO	5.00±0.00^A^	5.00±0.82^A^	5.00±0.82^A^

(B)				
Odor				
Methods	Treatments	0	28	56
C	C	5.00±0.00^A^	3.8±0.82^E^	3.1±0.55^H^
Brushing	Z10	5.00±0.00^A^	4.4±0.71^C^	3.8±0.45^F^
	Z10‐HEO	5.00±0.00^A^	4.7±0.45^B^	4.1±0.45^E^
	Z15	5.00±0.00^A^	4.5±0.55^C^	3.8±0.82^F^
	Z15‐HEO	5.00±0.00^A^	4.6±0.45^BC^	4.5±0.55^C^
	Z20	5.00±0.00^A^	4.5±0.82^C^	4.3±0.45^D^
	Z20‐HEO	5.00±0.00^A^	4.7±0.45^B^	4.5±0.81^C^
Dipping	Z10	5.00±0.00^A^	4.1±0.45^D^	3.5±0.71^G^
	Z10‐HEO	5.00±0.00^A^	4.7±0.55^B^	4.00±0.82^E^
	Z15	5.00±0.00^A^	4.4±0.71^C^	3.8±0.71^F^
	Z15‐HEO	5.00±0.00^A^	4.5±0.71^C^	4.2±0.45^DE^
	Z20	5.00±0.00^A^	4.6±0.55^BC^	3.8±0.55^F^
	Z20‐HEO	5.00±0.00^A^	4.6±0.45^BC^	4.5±0.55^C^
Spraying	Z10	5.00±0.00^A^	4.5±0.55^C^	3.6±0.45^FG^
	Z10‐HEO	5.00±0.00^A^	4.7±0.45^B^	4.00±0.82^E^
	Z15	5.00±0.00^A^	4.6±0.55^BC^	3.8±0.55^F^
	Z15‐HEO	5.00±0.00^A^	5.00±0.71^A^	4.1±0.45^E^
	Z20	5.00±0.00^A^	4.7±0.84^B^	4.5±0.72^C^
	Z20‐HEO	5.00±0.00^A^	5.00±0.82^A^	4.7±0.82^BC^
Enrobing	Z10	5.00±0.00^A^	4.5±0.82^C^	4.00±0.82^E^
	Z10‐HEO	5.00±0.00^A^	4.7±0.82^B^	4.5±0.45^C^
	Z15	5.00±0.00^A^	4.6±0.45^BC^	4.00±0.72^D^
	Z15‐HEO	5.00±0.00^A^	5.00±0.55^A^	5.00±0.55^A^
	Z20	5.00±0.00^A^	5.00±0.45^A^	4.8±0.55^B^
	Z20‐HEO	5.00±0.00^A^	5.00±0.45^A^	5.00±0.55^A^

(C)				
Overall acceptability				
Methods	Treatments	0	28	56
C	C	5.00±0.00^A^	3.5±0.72^G^	2.9±0.82^G^
Brushing	Z10	5.00±0.00^A^	3.8±0.81^F^	3.5±0.71^F^
	Z10‐HEO	5.00±0.00^A^	4.2±0.55^D^	3.7±0.71^EF^
	Z15	5.00±0.00^A^	4.5±0.45^CD^	3.8±0.45^E^
	Z15‐HEO	5.00±0.00^A^	4.6±0.55^C^	4.4±0.82^BC^
	Z20	5.00±0.00^A^	4.6±0.72^C^	4.3±0.55^C^
	Z20‐HEO	5.00±0.00^A^	4.7±0.55^BC^	4.1±0.71^D^
Dipping	Z10	5.00±0.00^A^	3.7±0.55^F^	3.4±0.71^F^
	Z10‐HEO	5.00±0.00^A^	4.00±0.82^E^	3.5±0.55^F^
	Z15	5.00±0.00^A^	4.00±0.55^E^	3.7±0.45^EF^
	Z15‐HEO	5.00±0.00^A^	4.1±0.55^DE^	3.7±0.71^EF^
	Z20	5.00±0.00^A^	4.2±0.71^D^	3.8±0.82^E^
	Z20‐HEO	5.00±0.00^A^	4.5±0.82^CD^	4.00±0.82^D^
Spraying	Z10	5.00±0.00^A^	4.00±0.82^E^	3.8±0.55^E^
	Z10‐HEO	5.00±0.00^A^	4.5±0.71^CD^	4.00±0.45^D^
	Z15	5.00±0.00^A^	4.5±0.45^CD^	3.8±0.82^E^
	Z15‐HEO	5.00±0.00^A^	4.7±0.55^BC^	4.00±0.55^D^
	Z20	5.00±0.00^A^	4.5±0.55^CD^	4.00±0.55^D^
	Z20‐HEO	5.00±0.00^A^	5.00±0.55^A^	4.5±0.55^B^
Enrobing	Z10	5.00±0.00^A^	4.5±0.45^CD^	4.1±0.45^D^
	Z10‐HEO	5.00±0.00^A^	4.8±0.45^B^	4.5±0.82^B^
	Z15	5.00±0.00^A^	4.8±0.55^B^	4.1±0.55^D^
	Z15‐HEO	5.00±0.00^A^	5.00±0.82^A^	5.00±0.82^A^
	Z20	5.00±0.00^A^	5.00±0.71^A^	5.00±0.71^A^
	Z20‐HEO	5.00±0.00^A^	5.00±0.55^A^	5.00±0.71^A^

*Note*: Different letters within the same day (A, B, C, etc.) indicate a statistically significant difference (*p* ≤ .05).

Abbreviations: C, control; HEO, *Heracleum persicum* essential oil; Z, Zein.

## 4 CONCLUSION

In the present study, the efficiencies of different coating techniques, including dipping, brushing, spraying, and enrobing methods by zein‐based edible coating containing HEO, were investigated on the shelf‐life of the whey‐less cheese. The enrobing method showed the highest efficiency in the shelf‐life enhancement of the cheese samples among the other techniques, which can be related to the integrity, uniformity, and thickness of the formed coating on the cheese surfaces. The spraying method formed a complete and rough coating on the cheese surfaces and could significantly (*p* ≤ .05) improve the quality of the cold stored cheese pieces after the enrobing technique. Brushing and dipping methods created an uneven and incomplete coating on the cheese surfaces that are placed in the third and fourth ranks in terms of performance, respectively. Also, the zein‐based edible coating could significantly (*p* ≤ .05) elongate the shelf‐life of the whey‐less cheese samples in a dose‐dependent manner compared with the uncoated samples. Furthermore, the addition of HEO to the zein biopolymer significantly (*p* ≤ .05) fortified the performance of all used coating methods in the shelf‐life improvement of the cold stored cheese samples. However, optimizing the effective factors (such as zein concentration, coating time, coating speed, nozzle dimension, and drying time) to control the uniformity and thickness of the coating and obtain the most appropriate and cost‐effective conditions is needed in the future.

## AUTHOR CONTRIBUTIONS

**Hadis Rajaei Lak:** Data curation (equal); formal analysis (equal); methodology (equal); software (equal). **Behnaz Bazargani‐Gilani:** Conceptualization (equal); data curation (equal); formal analysis (equal); investigation (equal); methodology (equal); supervision (equal); writing – original draft (equal); writing – review and editing (equal). **Mostafa Karami:** Conceptualization (equal); formal analysis (equal); investigation (equal); software (equal); validation (equal); visualization (equal).

## CONFLICT OF INTEREST STATEMENT

The authors declare no potential conflict of interest.

## Data Availability

The data that support the findings of this study are available from the corresponding author upon reasonable request.
